# Deferasirox Reduces Oxidative Stress in Patients With Transfusion Dependency

**DOI:** 10.4021/jocmr1180w

**Published:** 2013-01-11

**Authors:** Katsuyasu Saigo, Mari Kono, Yuri Takagi, Mariko Takenokuchi, Yasushi Hiramatsu, Hiroshi Tada, Terutoshi Hishita, Masahito Misawa, Shion Imoto, Shinsaku Imashuku

**Affiliations:** a Faculty of Pharmacological Sciences, Himeji Dokkyo University, 7-Kami-ohno, Himeji, Hyogo, Japan; b Cell Analysis Center, Scientific Affairs, Sysmex Corporation, 1-Murotani, Nisiku, Kobe, Japan; c Department of Hematology and Oncology, Himeji Red Cross Hospital, 1-Shimoteno, Himeji, Hyogo, Japan; d Department of Hematology, Himeji Medical Center, 68-Honmachi, Himeji, Hyogo, Japan; e Department of Hematology, Akoh Central Hospital, 52-Somoncho, Akoh, Hyogo, Japan; f Faculty of Health Science, Kobe Tokiwa University, 2-Otanicho, Nagata-ku, Kobe, Hyogo, Japan; g Department of Pediatrics, Takasago Seibu Hospital, 1-Nakasuji, Takasago, Hyogo, Japan

**Keywords:** Deferasirox, Oxidative stress, Ferritin, dROM, Neutrophil

## Abstract

**Background:**

Iron chelation therapy is useful against the over-accumulation of iron and is expected to reduce oxidative stress resulting from the Fenton reaction and Haber-Weiss reaction. We monitored oxidative status and serum ferritin levels after in vivo administration of deferasirox (DFS) and studied the in vitro effects of iron chelators on neutrophil function.

**Methods:**

Nine patients suffering from transfusion dependency were recruited for this study, and derivatives of reactive oxygen metabolite (dROM) tests to detect serum hydroperoxide levels were evaluated in addition to serum ferritin levels. Human neutrophil reactive oxygen species (ROS) production was determined with flow cytometry.

**Results:**

Ferritin levels decreased after DFS treatment (P = 0.068), and a significant reduction in dROM levels was measured (P = 0.031). Fifty microM DFS significantly inhibited ROS production induced by fMLP in vitro (P < 0.0001), and tended to inhibit that induced by PMA. On the other hand, deferioxamine failed to inhibit ROS production even at high concentrations.

**Conclusions:**

In vivo administration of DFS resulted in the reduction of oxidative stress, and this effect was considered to depend not only on a reduction in iron storage but also on the ability of DFS to inhibit neutrophil ROS production in vitro at clinically relevant plasma levels. Further studies are needed to examine the effects of iron chelators.

## Introduction

Transfusion dependency for chronic anemia causes excessive accumulation of iron and oxidative stress by the Fenton reaction and Haber-Weiss reaction, and several studies have reported that iron chelation therapy is useful against the over-accumulation of iron [1, 2]. Oral iron chelating agents have recently been introduced, and several studies have investigated the effects of iron chelation as well as its anti-oxidant or anti-tumor effects [2-4]. We used iron chelation therapy with deferasirox (DFS, Exjade^®^; Novartis-Pharma, Basel, Switzerland) in 9 patients with transfusion dependency resulting from hematologic disorders, and monitored their oxidative status and serum ferritin levels. DFS has also been proven to possess an anti-neutrophil reactive oxygen species production capability.

## Materials and Methods

Nine patients suffering from transfusion dependency (4 with myelodysplastic syndrome (MDS), 4 with aplastic anemia (AA), and one with multiple myeloma (MM)) were recruited between July 2008 and September 2010 for this study. Five males and 4 females with a mean age of 68.8 years (range: 45 - 80) were classified as transfusion dependent according to Malcovati’s criteria, where at least one RBC transfusion is performed every 8 weeks over a period of 4 months [5]. DFS was prescribed at a dose of 10 - 25 mg/kg and was sustained for at least 3 months. This study was approved by the Ethics Committee of Himeji Dokkyo University and written informed consent was obtained from all participants.

Serum ferritin levels were determined by means of the chemiluminescent immunoassay system Lumipulse^®^ (Fujirebio, Tokyo, Japan), and whole hemoglobin levels by one of two automatic hematology analyzers, XE-2100 (Sysmex Corp., Kobe, Japan) or Advia 2120 (Siemens, Berlin, Germany). Sera were stored at -80 °C and oxidative stress levels were determined by derivatives of reactive oxygen metabolite (dROM) tests using the FRAS4 system (Wismerll Co., Ltd, Parma, Italy) [6-10] to detect hydroperoxide levels relating to lipid, peptide, and amino acid oxidation. The coefficient of variation of dROM measured ten times using the serum of a volunteer was 5.4%.

Neutrophil reactive oxygen species (ROS) production was determined with flow cytometry using aminophenyl fluorescein (APF) (Sekisui Medical, Tokyo, Japan) as described previously [11, 12]. Mean fluorescent intensity (MFI) values after stimulation with 0.32 nM phorbol myristate acetate (PMA, Sigma-Aldrich, St. Louis, MO, USA) or 50 nM N-formyl methionyl leucyl phenylalanine (fMLP, Sigma-Aldrich) were compared in the presence or absence of preincubation with DFS or deferioxamine (DFO) (Both provided by Novartis-Pharma) for 30 min at RT.

Statistical analysis was performed with the JMP-7 package (SAS Institute Inc, Cary, NC, USA). The t-test was employed, and a P-value < 0.05 was considered significant.

## Results

Ferritin levels tended to decrease from 3,706 ± 2,558 (M ± SD) ng/mL to 2,443 ± 1,488 after DFS administration (P = 0.068). However, dROM levels decreased significantly from 409 ± 127 Carr U to 309 ± 65 (P = 0.031) (Table 1). Case 5, a patient with aplastic anemia, attained improvements in hemoglobin values, which was also seen in sporadic cases whose erythropoiesis recovered after deferasirox therapy [13].

**Table 1:** Clinical Data Before and After Deferasirox Treatment

Case no.	Diagnosis	Before deferasirox treatment			Duration	After treatment		
	age sex	ferritin (ng/mL)	dROM (Carr U)	Hb (g/dL)		ferritin	dROM	Hb
case 1	MDS 75F	1,971	603	4.5	5 Mo	1,031	349	4.5
case 2	MDS 67M	8,015	391	5.7	4 Mo	3,464	198	4.4
case 3	MDS 72M	2,669	232	7.9	8 Mo	1,357	260	6.6
case 4	MDS 80F	7,740	363	7.7	5 Mo	3,720	389	7.3
case 5	AA 80F	2,121	407	6.1	15 Mo	1,608	323	7.8
case 6	AA 73M	3,645	489	5.7	3 Mo	3,713	398	6.0
case 7	AA 52M	825	583	5.9	4 Mo	283	292	5.1
case 8	AA 45F	4,200	299	6.8	4 Mo	4,639	258	6.7
case 9	MM 75M	2,172	317	7.9	8 Mo	2,172	311	6.2
mean		3,706	409	6.5		2,443	309	6.1
SD		2,558	127	1.2		1,488	65	1.2

MDS: myelodysplastic syndrome; AA: aplastic anemia; MM: multiple myeloma; Hb: hemoglobin.

**Figure 1:**
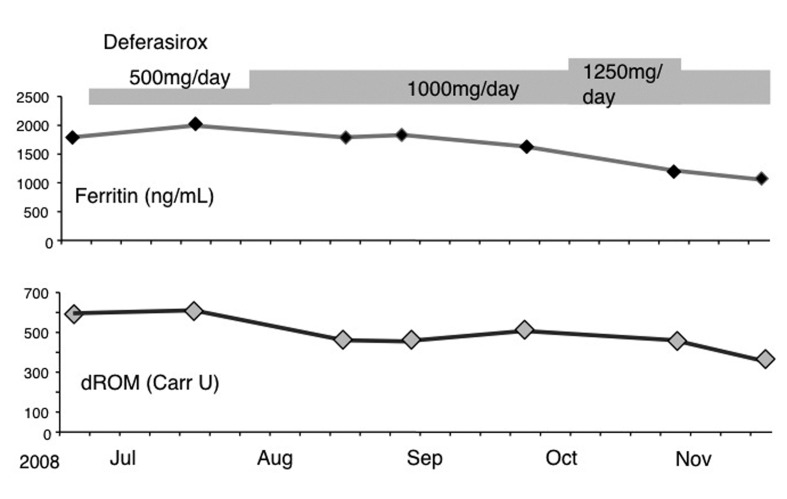
Clinical course of case 1.

Figure 1 shows the clinical course of case 1, an MDS patient, who exhibited a parallel regression in ferritin and dROM levels, suggesting a relationship between the two. In this case, the frequency of transfusion requirements did not change. When mild renal insufficiency developed during DFS administration at 1,250 mg/day, the daily dosage was reduced so that the treatment could be continued.

**Figure 2:**
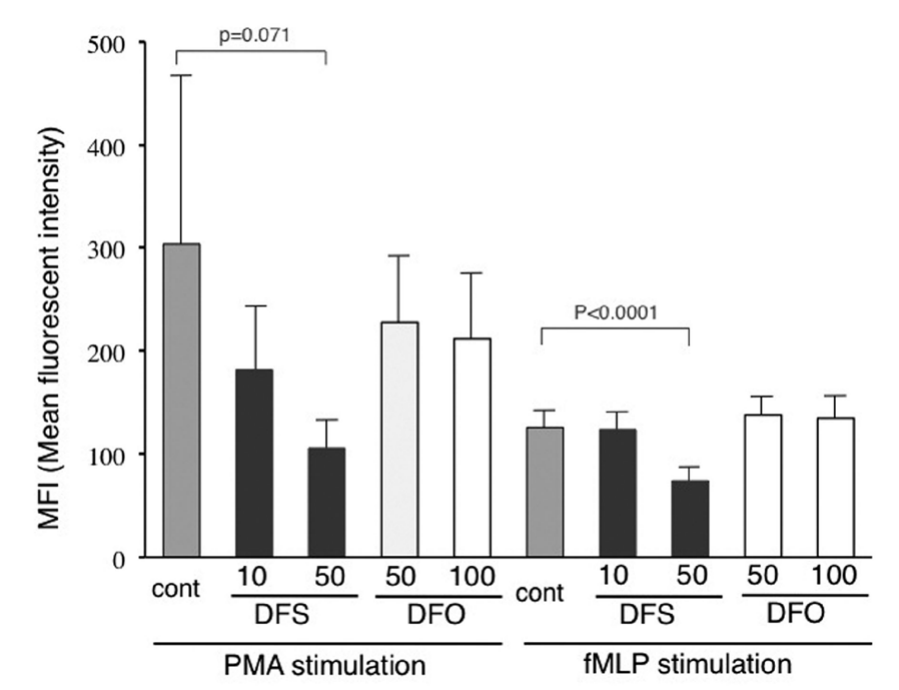
Effects of DFS and DFO on neutrophil ROS production. ROS production was detected by flow cytometry and results are shown in terms of mean florescent intensity (MFI). DFS was used at 10 and 50 μM, with DFS at 50 and 100 μM as final concentrations. Statistical analysis was performed with the t-test. Neutrophils were preincubated with iron chelators and then stimulated with fMLP or PMA (DFS: n = 8; DFO: n = 4).

We measured the direct anti-oxidant effects of DFS on human neutrophils, and, as shown in Figure 2, 50 microM DFS significantly inhibited ROS production induced by fMLP, and also tended to inhibit that induced by PMA. On the other hand, DFO did not suppress ROS production from neutrophils.

## Discussion

The over-accumulation of iron in humans causes oxidative stress resulting from the Fenton reaction and Haber-Weiss reaction. In patients with transfusion dependency, severe anemia itself induces oxidative stress [6, 9] and reduces hepcidin production [14], resulting in an increase in iron absorption accompanied by oxidative stress. As oxidative stress is reportedly one contributor to the pathophysiology of MDS [15], iron chelation therapy is thought to be effective in breaking this cycle. A newly developed oral iron chelator, DFS, is reportedly effective not only for iron chelation but also for reducing oxidative stress [2, 15, 16]. It was also previously shown that DFS reduced both labile plasma iron and labile cell iron more effectively than DFO in a primary and established cell line of cardiomyocytes [17].

Although the reduction in ferritin levels was not significant, dROM levels decreased significantly after DFS treatment, suggesting the presence of other mechanisms of DFS affecting redox status irrespective of iron chelation. It has been reported that iron chelators have anti-oxidant properties [3, 18]; however, there have been no reports on the effects of DFS on neutrophil function, including ROS production. Our in vitro study using flow cytometry demonstrated that DFS has the ability to inhibit neutrophil ROS production at clinically relevant plasma levels. On the other hand, DFO failed to inhibit ROS production even at high concentrations, although one study observed the inhibitory effects of DFO using a chemiluminescence method that detects extracellular ROS [19]. This finding may be related to the fact that DFO has a larger molecular weight and, unlike lipophilic DFS, is hydrophilic, which suggests that cellular membranes may be more permeable to DFS. Moreover, Messa et al reported that DFS has the unique property of NF-kappa B inhibition, which has not been observed in any other iron chelators [3].

In conclusion, DFS can reduce oxidative stress as well as iron chelation. This effect seems to be dependent on several mechanisms such as iron chelation, which reduces labile iron, inhibition of ROS production from neutrophils, and changes in signal transduction mechanisms. Further studies are thus needed to examine the general and specific effects of iron chelators on redox status and cellular functions, in addition to those on iron chelation.
